# UAVs for Structure-From-Motion Coastal Monitoring: A Case Study to Assess the Evolution of Embryo Dunes over a Two-Year Time Frame in the Po River Delta, Italy †

**DOI:** 10.3390/s19071717

**Published:** 2019-04-10

**Authors:** Yuri Taddia, Corinne Corbau, Elena Zambello, Alberto Pellegrinelli

**Affiliations:** 1 Engineering Department, University of Ferrara, Saragat 1, 44122 Ferrara, Italy; alberto.pellegrinelli@unife.it; 2 Physics and Earth Science Department, University of Ferrara, Saragat 1, 44122 Ferrara, Italy; corinne.corbau@unife.it (C.C.); elena.zambello@unife.it (E.Z.)

**Keywords:** coastal mapping, coastal monitoring, Digital Elevation Models (DEMs), geomorphological evolution, photogrammetry, Structure-from-Motion (SfM), Unmanned Aerial Vehicles (UAVs)

## Abstract

Coastal environments are usually characterized by a brittle balance, especially in terms of sediment transportation. The formation of dunes, as well as their sudden destruction as a result of violent storms, affects this balance in a significant way. Moreover, the growth of vegetation on the top of the dunes strongly influences the consequent growth of the dunes themselves. This work presents the results obtained through a long-term monitoring of a complex dune system by the use of Unmanned Aerial Vehicles (UAVs). Six different surveys were carried out between November 2015 and December 2017 in the littoral of Rosolina Mare (Italy). Aerial photogrammetric data were acquired during flight repetitions by using a DJI Phantom 3 Professional with the camera in a nadiral arrangement. The processing of the captured images consisted of the reconstruction of a three-dimensional model using the Structure-from-Motion (SfM). Each model was framed in the European Terrestrial Reference System (ETRS) using GNSS geodetic receivers in Network Real Time Kinematic (NRTK). Specific data management was necessary due to the vegetation by filtering the dense cloud. This task was performed by both performing a slope detection and a removal of the residual outliers. The final products of this approach were thus represented by Digital Elevation Models (DEMs) of the sandy coastal section. In addition, DEMs of Difference (DoD) were also computed for the purpose of monitoring over time and detecting variations. The accuracy assessment of the DEMs was carried out by an elevation comparison through especially GNSS-surveyed points. Relevant cross sections were also extracted and compared. The use of the Structure-from-Motion approach by UAVs finally proved to be both reliable and time-saving thanks to quicker in situ operations for the data acquisition and an accurate reconstruction of high-resolution elevation models. The low cost of the system and its flexibility represent additional strengths, making this technique highly competitive with traditional ones.

## 1. Introduction

Beaches are usually characterized by vegetated dune systems performing a variety of functions valuable to the ecosystem, such as protection against the ingression of the sea water during storms and a sand reservoir that supplies sand to eroded beaches during storms and buffers windblown sand and salt spray. In addition, the dunes act as an ecological niche for both flora and fauna.

In a natural context, coastal dunes can be classified into three main types: incipient foredunes, established foredunes, and foredune plains [1,2]. The incipient foredunes, or embryo dunes [3], are low dunes formed by aeolian sand deposition within pioneer plant communities on the backshore of beaches [2,4]. Their formation is due to an increase in surface roughness due to the presence of some elements on the surface of the backshore responsible for a reduction of wind flow velocities, resulting in sediment deposition.

From a geomorphological point of view, the embryo dunes present the most interesting shapes to be assessed because they are particularly sensitive to even the smallest changes in any of the coastal environment factors. Indeed, they can quickly be destroyed as they are created. Due to this importance, the presence of embryo dunes hence represents a crucial parameter for the final selection of the case study location.

**Figure 1:**
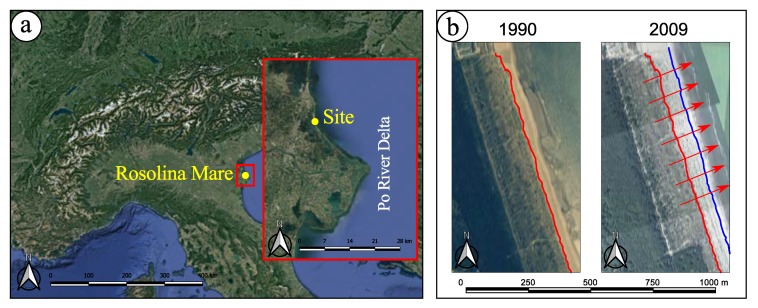
(**a**) The case study location, northern Italy (by Google Earth); (**b**) The progression (*progradation*) of the embryo dunes’ limit over 19 years in the past.

Consequently, the monitored site (Figure 1a) presents all the typical characteristics of a natural dune system previously mentioned. It is located in the northern Adriatic Sea close to the Po River delta (Italy), near the town of Rosolina Mare. The beach of the monitored area extends up to 300 m in the North-South direction. The back-dunes environment is characterized by the presence of several infra-dune depressions in which the rainwater is collected. That allows the growth of a particular hygrophilous vegetation. A patch of bushy plants covers the innermost established foredunes and spatially precedes the arboreal formations. The forest is made of both autochthonous holm oaks and pines of artificial origin.

Moreover, the inclusion of this coastal stretch within the “Coastal Botanical Gardens” of Porto Caleri ensures both the complete conservation and the natural evolution of the monitored coastal environment. No significant impact of human activities has been noticed during the last few years preceding the beginning of the surveys; hence the site was found to be particularly suitable for the assessment of the natural geomorphological evolution of dunes, focusing on the embryonic stage.

During recent decades, all the southern part of the Rosolina Mare littoral experienced a significant progradation of the shoreline. Figure 1b highlights the progression of the embryo dunes’ limit from 1990 to 2009 as it was observed by aerial orthophotos and satellite imageries. In fact, survey techniques based on remote sensing from satellite were successfully used in the past to assess these phenomena by some authors [5]. The overall progression detected at Rosolina Mare was of about 50 m over almost 20 years while a maximum rate of 5 m per year was noticed. The progradation is still active at the site selected for this work where the embryo dunes generally grow and join each other, due to the aeolian transport [6].

The reconstruction of Digital Elevation Models (DEMs) represents a primary tool to detect and characterize the morphology of complex systems. Their comparison enables an accurate assessment, especially when the shapes change rapidly in both time and space. Different techniques are currently available for generating DEMs at a variable spatial resolution. Using discrete approaches, a large amount of points may be directly surveyed through GNSS geodetic receivers. When carried out by means of a Network Real Time Kinematic (NRTK) technique, the survey is hence already framed in an official reference system. Alternatively, the same detection can be done in a local reference system by using a total station. Regardless of the instrumentation used, the manual mapping of the shapes is a very time-consuming task and cannot be applied to broad extensions.

Further approaches, with a dense (and almost continuous) data collection, such as Terrestrial Laser Scanners (TLSs), airborne LiDAR (Light Detection And Ranging) and digital photogrammetry by Unmanned Aerial Vehicles (UAVs), enable the remote sensing of wide areas, still with a good level of accuracy. LiDAR has been perhaps the most common technique used for the purpose of mapping long coastline sections. In fact, the raw data acquired by this technique have high spatial resolutions. However, the subsequent data processing is usually imprinted to provide a resampled model to the users, characterized by a Ground Sample Distance (GSD) of about 0.5–1 m. Moreover, neither the characteristics of the instruments used to acquire the raw data nor the processing procedures (filtering and georeferencing techniques) further applied to them to generate the final products (clouds, DEMs) are actually known for all the LiDAR datasets currently available in the Rosolina Mare area. Despite this particular issue, the LiDAR approach is certainly an effective method for monitoring broad extensions. However, the sandy coastal environment evolves too much rapidly to be assessed by airborne LiDAR recognitions and the alternative use of helicopters has prohibitive costs. For advanced studies in coastal geomorphology, a different method with a great flexibility and able to provide better spatial resolutions at a more reasonable cost is hence needed.

A solution is represented by the recent availability of lightweight and compact digital cameras along with the continuous development of newer and more powerful algorithms in computer vision. In addition, the use of small UAVs has revolutionized the classical aerial photogrammetry leading to a new digital photogrammetry based on a Structure-from-Motion (SfM) approach that provides excellent results in terms of spatial resolution at a low cost [7].

**Figure 2:**
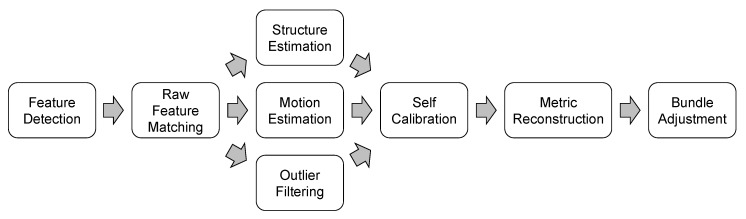
The Structure-from-Motion workflow.

The SfM process (Figure 2) is an advanced method of the computer vision branch. This approach enables the three-dimensional geometry reconstruction (the so-called *structure*) starting from a set of two-dimensional images of a scene (the so-called *motion*). The first stages are the detection of features on each image, their characterization through descriptors and the final matching of the established correspondences. The resulting matches, i.e., the tie points, constitute a sparse point cloud: this is a first raw model of the detected object. It is important noticing that no *a priori* assumption, such as the use of Ground Control Points (GCPs), is needed to reconstruct the geometry, since the well-known collinearity equations [8] can be solved in any arbitrary scale. A further advantage of the SfM lies in the concurrent estimation of both the exterior and interior orientation of the camera, performing the so-called *self-calibration* [9]. This is particularly useful whenever an uncalibrated camera is used for the acquisition of aerial imageries. Both the symmetric and asymmetric radial distortions can be modelled through a self-calibration.

To perform an accurate georeferencing of the point cloud model, a set of GCPs was surveyed by GNSS geodetic receivers through NRTK. The accuracies of such a technique are completely comparable with the Root Mean Square Error (RMSE) of a 2 cm GSD imagery in a SfM approach [10]. To frame the model in the same reference system of GCPs, a final block bundle adjustment (BBA) procedure was adopted [11], performed within the same software used to reconstruct the geometry (Agisoft PhotoScan Professional). BBA can account for all the non-linearities, hence it provides better results than adopting a simpler similarity transformation. Moreover, a wide distribution of GCPs can help to prevent any possible bowl-effect on the model due to nadiral imageries, since no oblique images that may have minimized this issue [12] were ever captured in this work.

Once the three-dimensional geometry has been reconstructed thanks to the tie points detection, a point cloud with higher density can be generated using specialized algorithms capable to reconstruct depth maps and searching for additional features to be matched. For this reason, the final dense cloud represented the starting-point for any further data processing. On average, each dense point cloud in this work contained up to ca. 80 million points.

There are increasingly reports in the literature regarding applications of UAV systems, also for the aim of a coastal monitoring [13]. Gonçalves and Henriques [14] studied a similar sandy coastal environment, while more recent applications regarding the Adriatic littoral have been shown by Scarelli et al. [15] in the Emilia-Romagna region. Taddia et al. [16] illustrated a very first approach about embryo dunes surveying techniques, but without showing the results of a long-term assessment. In addition, other authors also tested the SfM approach by UAV assessing the vertical accuracy of the products compared to TLS technology [17] or investigated the accuracy of georeferenced point clouds produced via multi-view stereopsis from UAV imagery flying at an altitude very close to ours [18]. All these studies confirm the reliability of the use of UAV-acquired imageries in coastal environments.

The SfM by UAVs therefore represents an innovative technique able to speed up the acquisition process and it finally provides accurate and reliable products. These considerations, along with its versatility, low cost, effectiveness [19] and significantly higher spatial resolutions than other methods, led us to adopt the SfM by UAV as remote sensing approach in the context of geomorphological coastal monitoring.

The innovative aspect of the present study lies in the use of the SfM technique not only for the simple mapping of a coastal section, but instead for the repetition of the survey with the purpose of monitoring the embryo dunes’ morphological evolution. Indeed, we tested the applicability of these methodologies in the study of morphological changes of a dune system due to natural factors on a seasonal time scale. In particular, we will highlight the accuracy of the UAV-borne surveys and quantify incipient foredune morphodynamics. Such results may also contribute to the application of UAV surveys in coastal management studies.

## 2. Materials and Methods

The characterization of the dune system morphology was performed using UAVs [20], in particular for the detection of the embryo dunes with a small size. Indeed, LiDAR resolution, especially whenever resampled at a 0.5–1 m GSD, is definitely unsuitable for this latter purpose [21]. As previously mentioned, the decision to adopt SfM by UAVs was dictated by the great potential of the technique itself, including a fast image acquisition at a low cost and the ability to generate very high-resolution products (orthomosaics, DEMs) [22].

**Figure 3:**
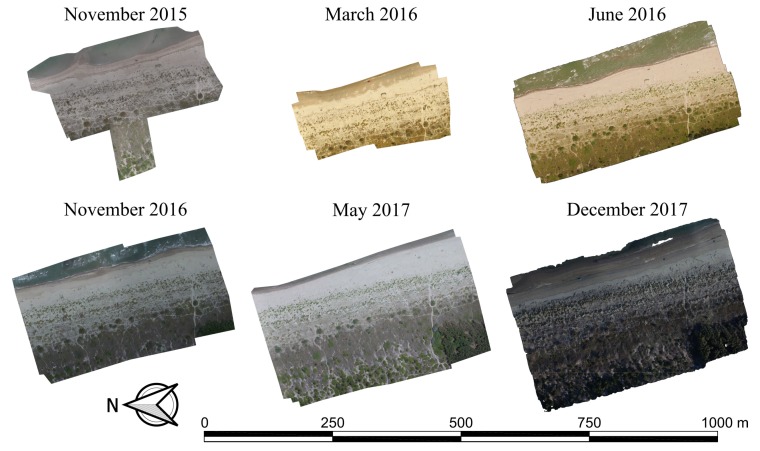
Orthomosaics of the six different survey repetitions from November 2015 to December 2017 showing the extent of the surveyed regions.

**Figure 4:**
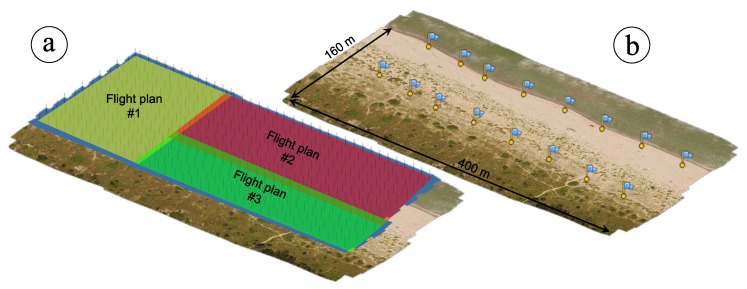
(**a**) Different flight plans’ coverage with overlap for including all the study area; (**b**) Ground Control Points location and extent of the site.

Six survey repetitions were carried out respectively on November 2015, March 2016, June 2016, November 2016, May 2017 and December 2017 (Figure 3). For each flight, a specific planned mission was set up. Several flights between two and three (Figure 4a) per repetition was necessary to map the full extent of the monitored area due to both the limited autonomy of the UAV (related to battery capacity) and the need to maintain an altitude of about 40 m. That was necessary to achieve the desired spatial resolution of 2 cm.

**Figure 5:**
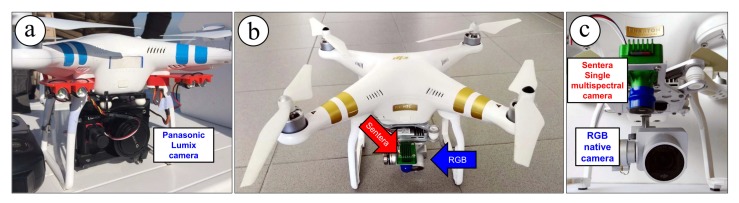
(**a**) The DJI Phantom 2 Vision equipped with the Panasonic Lumix camera; (**b**) The DJI Phantom 3 Professional equipped with both the RGB native camera (highlighted in blue) and a Sentera Single multispectral sensor (highlighted in red); (**c**) A detail on both the DJI Phantom 3 cameras, showing the nadiral fixed arrangement of the Sentera Single sensor.

Two aircraft were used: a DJI Phantom 2 Vision and a DJI Phantom 3 Professional, even though the DJI Phantom 2 was actually used for the first survey only (on November 2015), whereas the DJI Phantom 3 was used for all the subsequent survey repetitions. They were equipped respectively with a Panasonic Lumix DMC-GM1 camera (16-megapixel RGB sensor) and a DJI FC300X camera (12-megapixel RGB sensor). Specifications of aircraft and cameras are listed in Table 1. The main reason for using the DJI Phantom 3 was that it was stably equipped with a low-cost RGNIR sensor (Sentera Single) with the capability to acquire information in the near infrared (NIR) wavelengths in an independent way with respect to the DJI FC300X camera (Figure 5). The availability of an additional spectral information (potentially to be used for further analysis or processing) argued for using the DJI Phantom 3 aircraft.

Thanks to the ability of the combination of SfM and BBA to model any kind of uncalibrated camera through a self-calibration procedure with a very good estimation of all interior parameters and lens distortion, especially whenever a redundant set of images is available, the differences induced by using different cameras on different aircraft were negligible.

Generally, the flight plans parameters were imposed as shown in Table 2. The camera was always in a nadiral arrangement. Some camera settings (e.g., exposure time) were varied according to the real brightness of the scene at the time of each flight: in this case a range of values is given.

Each survey was framed within the European Terrestrial Reference System (ETRS) horizontal datum, in its ETRF 2000 (2008.0) realization. The National Dynamic Network (RDN) materializes this reference system in Italy, thanks to the same Continuously Operating Reference Stations (CORSs) used to stream corrections for the NRTK approach. Targets were distributed over the ground to almost completely enclose the surveyed area (Figure 4b) and were thus used as Ground Control Points. Generally, an amount between 14 and 18 was deployed. Each target, designed to be easily recognized on the images, with a clear and unmistakable position of its center, was surveyed with a GNSS geodetic receiver in NRTK mode. The accuracy achievable with a kinematic “stop and go” technique, with coordinates collected averaging 30 s of observations and corrections transmitted in real time from a network of CORS (the ItalPoS service was used for this purpose), is sufficient and comparable with the RMSE of a 2 cm GSD SfM-derived model. In addition, the need for any post-processing of the GNSS collected data for the final computation of the targets’ coordinates is suppressed in this way. All the elevations were framed within the Italian vertical datum by applying the ITALGEO 05 geoidal separation model to convert ellipsoidal heights to orthometric elevations.

**Figure 6:**
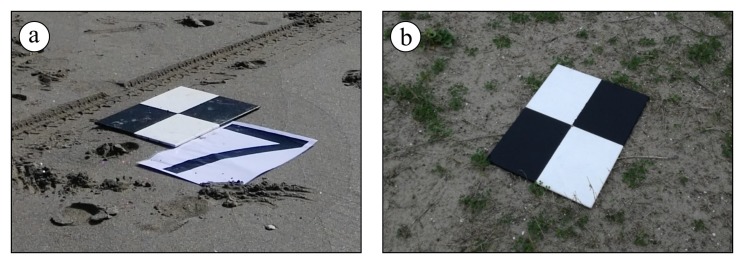
Targets used as Ground Control Points to be recognized on the aerial images placed (**a**) on the shoreline (initially numbered to be used for the processing of the Panasonic Lumix DMC–GM images) and (**b**) on the back-dunes (not numbered thanks to the Exif metadata in the DJI FC300X images).

The need to number each GCP (Figure 6) to be recognized on the images was overcome thanks to the GPS Exif metadata in the images acquired with the DJI Phantom 3 native camera. Indeed, relative distances from a target to the others were much higher than the absolute georeferencing error of a first raw alignment carried out based on the Exif camera locations. This implied that the closer target reprojected on an image was also the correct one to be specified on the image itself. The recognition of the targets on each image was thus performed manually: this operation was not particularly complex thanks to the mentioned reprojection of a marker once the target has been placed on two aligned images at least.

A second alignment was performed once all the GCPs were identified on the images. In this latter alignment, all the camera locations were ignored due to their very poor accuracy. Thus, they were assigned with a very low weight or even simply deleted. A final optimization for reprojecting was performed after this second and final alignment in order to best fit the interior orientation parameters of the camera (focal length *f*, principal point’s coordinates $c_{x}$, $c_{y}$, radial distortion coefficients $k_{1}$, $k_{2}$, $k_{3}$, tangential distortion coefficients $p_{1}$, $p_{2}$) and to achieve the maximum accuracy of the 3D model. A summary of residuals for each survey repetition is shown in Table 3.

**Figure 7:**
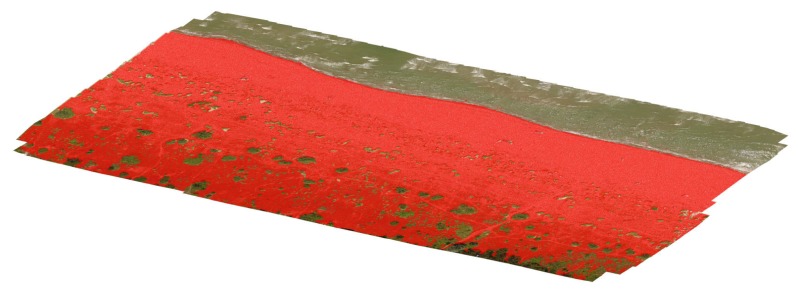
Dense point cloud after the detection of the ground points by the use of the slope-based classification algorithm in Agisoft Photoscan Professional.

**Figure 8:**
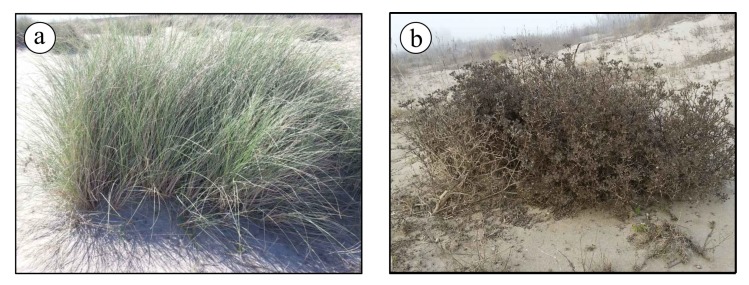
Botanical species of vegetation on the dunes: (**a**) *Ammophila arenaria*; (**b**) *Echinophora spinosa*.

The successful reconstruction of the three-dimensional geometry based on the matched features (tie points) was then followed by the creation of a dense cloud. This task has been performed using the same software used for the alignment of the images (Agisoft PhotoScan Professional), through the means of robust densification algorithms already implemented in it. However, the dense cloud represents a model of the outer surface of the detected object, therefore it contains points belonging to the vegetation on the top of the dunes instead of the sand at their bases. This means that it is not helpful to detect and assess any morphological change of the embryo dunes over time. To remove those points from any further data processing (including the creation of DEMs) a filter on a slope detection was used. The filter is a feature already available within Agisoft PhotoScan and it performs a ground points classification. Essentially, it [23] works through two steps: in the first one, the entire dense cloud is divided into several square cells of a user-specified size. The value adopted in this work 5 m × 5 m. The detected lowest point of each cell is hence assumed to belong to the ground and a first raw model of the ground itself is then created using the lowest points from all the cells. In the second step, all other points are classified by detecting both their distance and slope from the ground-classified points of step one. For this further task, threshold values for distances and slopes must be specified. In this work we adopted respectively 0.15 m and 15°. The final dense cloud of ground-classified points is shown in Figure 7, where all the points classified as effective ground are highlighted in red. Examples of botanical species found in the dune system and responsible for this issue are *Ammophila arenaria* (Figure 8a) and *Echinophora spinosa* (Figure 8b).

**Figure 9:**
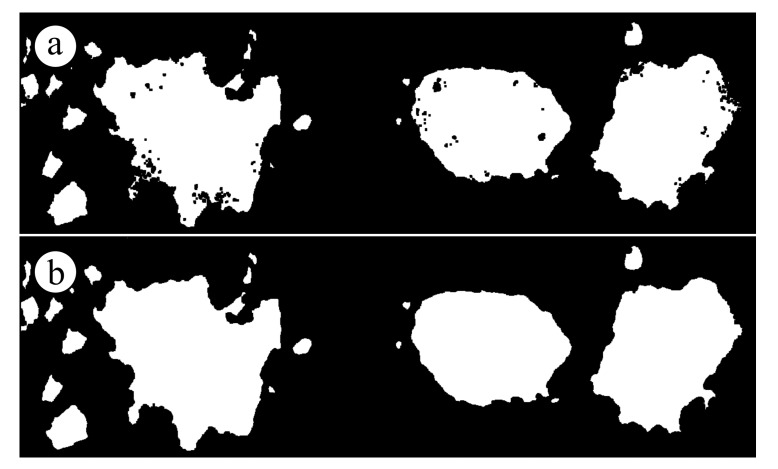
(**a**) Quality of the dense cloud after the detection of ground points on a detail. Some outliers are still present; (**b**) Quality of the dense cloud after the application of the statistical outlier removal (SOR) filter on the same detail. Outliers have been removed.

Since outliers were still present after the classification, a further statistical outlier removal (SOR) filter was applied to the dense cloud to remove residual points before proceeding with the creation of a DEM of the sandy terrain. This task was performed using the CloudCompare software. Figure 9 shows the effect of the SOR filter in detail: on the top (Figure 9a) a zoom on the raw dense cloud of ground-classified points, on the bottom (Figure 9b) the same region after the application of the filter, showing the effective outliers removal. The values for the parameters to set in such a filter were carefully assumed each time to maximize the filtering effect. Figure 9 was obtained assuming 50 points for the mean distance estimation and 1.5 as the standard deviation multiplier. After the removal of the residual vegetation outliers, a set of special NRTK surveyed points was then added to the filtered dense cloud to integrate all the regions where the ground data was missing.

**Figure 10:**
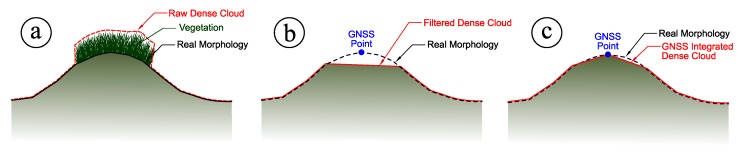
Schematization of the GNSS integration process by (**a**) filtering out the vegetation; (**b**) the survey of a point by GNSS and (**c**) the subsequent reconstruction of a more faithful shape.

**Figure 11:**
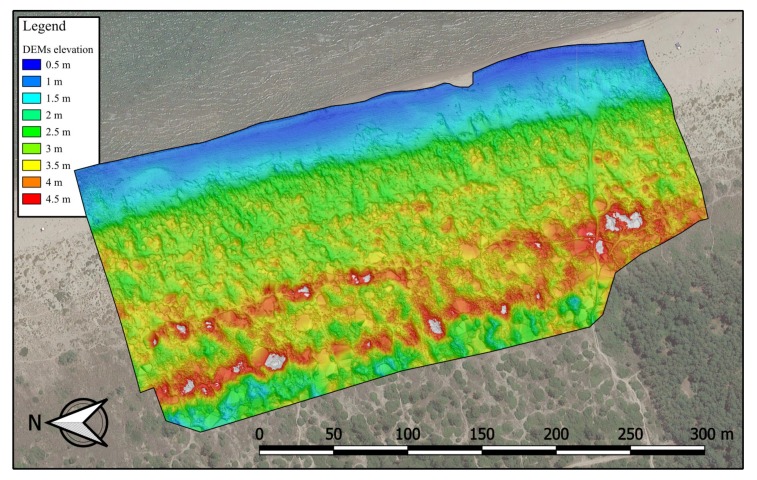
Digital Elevation Model (DEM) of the survey carried out on November 2016.

Such a process, from the raw dense cloud to the final integration with GNSS-acquired information, is shown in Figure 10 and it is summarized in the following. The raw dense cloud profile (Figure 10a) was eliminated by the application of the slope detection-based algorithm, thus obtaining a classification of the solely ground points (Figure 10b). Finally, an integration with specially surveyed GNSS points was performed to reconstruct a more faithful profile of the dense cloud itself (Figure 10c). The final GNSS-integrated dense point cloud was hence rasterized creating the DEM (Figure 11) Due to the very high density of points was so high (up to 1250 points per square meter), the simple average of the in-cell points’ height was assumed as the elevation for each DEM’s pixel, making it unnecessary to create a Triangulated Irregular Network (TIN) as intermediate step.

**Figure 12:**
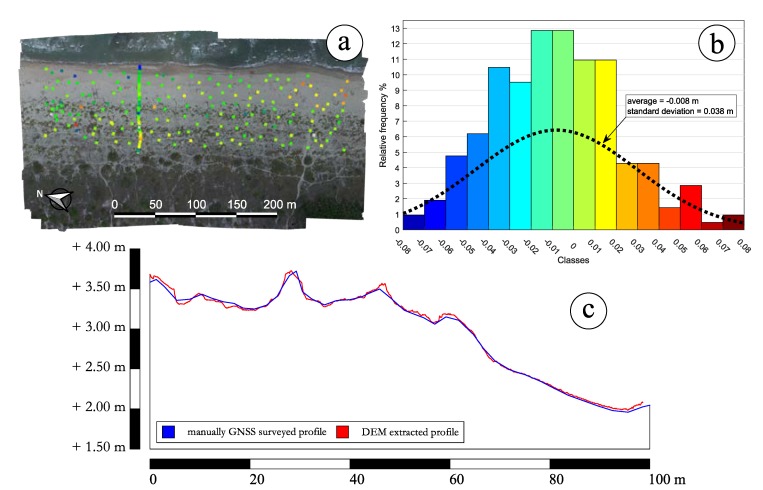
(**a**) Set of ground points used for the comparison between the DEM of November 2016 and the GNSS-surveyed elevations; (**b**) Validation results by histograms: the standard deviation value is comparable with the assumed accuracies of the SfM approach with a 2 cm GSD; (**c**) Comparison between GNSS and DEM on a profile. Discrepancies are still comparable with the SfM approach.

The DEM of each survey repetition was thus preventively checked [24] by comparing the model’s elevations with another set of GNSS-surveyed points (Figure 12a). Their coordinates were collected independently from those points used for the integration process described above. Moreover, a particular care was adopted when surveying directly on the sand, e.g., by using a plate to prevent the pole tip from sinking, which could have been a remarkable source of errors. The possible presence of systematic errors was also accurately investigated and statistically assessed (Figure 12b). Indeed, this check was used to validate the real accuracy of the DEM with respect to the actual morphology of the dunes system. The validation results for the DEM on November 2016, which represents the survey in which the widest set of validation points was collected, are shown in Figure 12. Both the average and the standard deviation values reconfirm the full compatibility between the SfM approach and the NRTK method of surveying used to reference the model. Similar values, even if not explicitly reported, were obtained for the validation of the DEMs generated for the other repetitions.

For a further and last validation, a cross-section profile was also reconstructed through GNSS NRTK during the solely survey on November 2016, to both assess the quality of the profiles reconstructed by a DEM extraction and the effectiveness of the plate under the pole. Figure 12c shows the results of such a comparison between this profile and its DEM derived one. Except for some outliers where the influence of vegetation was still slightly present, the computed average value of −1.2 cm for the difference between DEM (red line) and GNSS profiles (blue line) shows, once again, that the accuracy of the DEM is the same achievable by the use of the NRTK technique that was used for providing the vertical datum to the model.

## 3. Results

Once successfully performed the validation of each computed DEM generated, with discrepancies similar to those mentioned above, a series of further information was finally extracted from the DEMs and then compared each other. In fact, the strength of the aerial imageries by UAVs, with respect to any traditional discrete and time-consuming approach based on the use of total stations or GNSS geodetic receivers in NRTK mode, lies in the possibility to make cross sections in any region of the model at any time, thanks to a practically continuous data.

**Figure 13:**
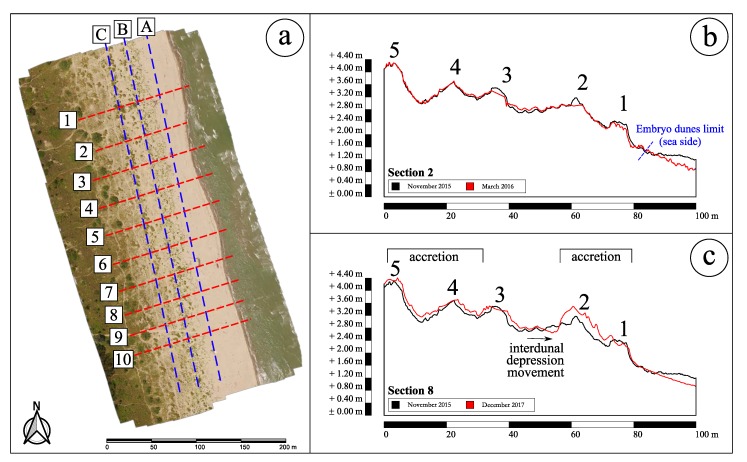
(**a**) Location of the cross and longitudinal sections; (**b**) Detection of the morphological evolution of dunes during first winter season by the comparison of the profiles of November 2015 (black) and March 2016 (red) on cross-section 2; (**c**) Detection of the morphological evolution of dunes over two years by the comparison of the profiles of November 2015 (black) and December 2017 (red) on cross-section 8.

In particular, to monitor the changes in the morphology of the studied coastal area, ten cross sections and three longitudinal sections (Figure 13a) were used to extract profiles.

Figure 13b illustrates a profile analysis conducted on the cross-section 2 to evaluate the effects of the first winter season. Cross-shore profile changes highlight the effects of the occurred winter sea-storms (November 2015 to March 2016). On this profile, five dunes may be observed. Dunes 5 and 4 appear stable, dunes 3 and 2 are characterized by a crest erosion while sediment deposition occurred in the interdune between 3 and 2. Finally, the seaward side of dune 1 and the backshore are in erosion.

**Figure 14:**
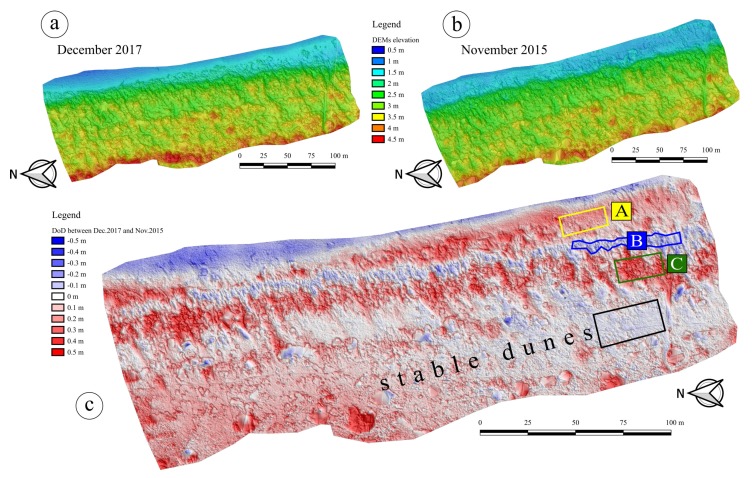
(**a**) DEM of the survey on December 2017; (**b**) DEM of the survey on November 2015; (**c**) Computation of the DEM of Difference (DoD) as $H_{Dec.2017}^{ortho}-H_{Nov.2015}^{ortho}$. Polygons describing stable dunes, zone *A*, zone *B* and zone *C* have been specified.

Moreover, an elevation difference was computed generating a DEM of Difference (DoD) for the whole extent of the overlapping area between different surveys. This enabled to compute a sediment budget through the compared area extents in a simple pixel per pixel computation, exploiting the accurate georeferencing of every DEM. Figure 14 shows the elevation differences between the last and the first survey, respectively performed in December 2017 and November 2015. The expression of the computed quantity is given by $H_{Dec.2017}^{ortho}-H_{Nov.2015}^{ortho}$, since the orthometric height was used. Red zones represent accumulation regions (positive sediment budget), while blue zones represent erosion areas (negative budget).

Furthermore, Figure 13c shows the evolution of the cross-section 8 during all the two-year period (November 2015 to December 2017), which is still characterized by the presence of five foredunes. The evolution of the dune system is positive with height variations reaching +0.40 m. Locally, erosion is also observed and in particular on the interdunal depression and on the upper backshore. We may also observe that foredunes 1 and 2 merged resulting in a higher foredune (about +0.20÷0.40 m).

The analysis of the DoD in Figure 14c thus shows how the progradation phenomenon is clearly detectable through two years of monitoring. In particular, the back-zone is stable. Zone *A* reported in Figure 14 represents the latest developed embryo dunes, while zones *B* and *C* indicate a seaward displacement of the dune system. Indeed, zone *B* is lower than two years before while zone *C* represents the area where the interdunal depression itself has been filled. Also, the cross-section in Figure 13c helps to better understand both the ongoing progradation and the interdunal depression progression. The comparison between profiles over two years hence confirms what DoD shows: a general increase of elevations that is the proof of a progradation phenomenon that is still active in the Rosolina Mare region where the site was selected.

Even though a thorough geospatial analysis is certainly beyond the aim of this work, in order to further support the DoD results some basic statistics have been computed on the previously highlighted regions: stable dunes, zone *A*, zone *B* and zone *C*. For each of these regions, a polygon was selected to be the most representative as possible (shown in Figure 14c). Nonetheless, high standard deviations of the computed elevation differences within the polygons may be due not only to an actual noise on the input DEMs, but they also might be caused by a slight heterogeneity of the region itself. Table 4 summarizes pixel counts, mean values, standard deviations and min/max values extracted from the DoD on each region.

It is worth noting that the stable dunes’ region shows a mean value close to zero, with a discrepancy (−0.029 m) that is comparable to the 0.046 m standard deviation. This fact is in accordance with the very low rate of evolution of stabilized back-dunes, especially if assessed over a two-year period only. Conversely, zones *A* and *C* are characterized by significantly positive mean values (respectively +0.229 m and +0.359 m) and thus they describe an actual ongoing deposition caused by the aeolian transport. Again, this fact confirms the high dynamics of the dune system seaward. Min/max values point out that few outliers are still present in the stable dunes’ region despite the performed data filtering and that the polygons of the other zones not being perfectly homogeneous. Similarly, the negative mean value computed in zone *B* also shows a real interdunal depression progradation with a significant lowering of the beach in those intermediate areas.

From a geomorphological point of view, the definition of control regions such as the ones selected above may be useful for the purpose of monitoring the progradation of the dune system over time, as well as to assess the formation of new embryo dunes near the shoreline.

## 4. Discussion

DEMs represent one of best procedures to detect complex system’s morphology, both in terms of high spatial resolution and accuracy. Presently, different techniques can collect data to be processed for finally obtaining DEMs. However, many are the aspects to be considered to adopt the most suitable approach, both in terms of expected results and costs.

The need for both high spatial and temporal resolutions to assess the evolution of critical environments subjected to rapid morphological changes, such as complex dune systems in coastal environments, can be certainly achieved with a SfM approach using UAVs as tools for the acquisition of the aerial imageries. This kind of technique represents an excellent compromise between the accuracy and the resolution typical of laser scanning systems with a very low cost, making it possible to repeat surveys also in shorter time intervals. Hence, the SfM approach can be successfully used to reconstruct any three-dimensional geometry. Moreover, it can model both the interior and exterior orientation parameters of the camera, allowing the use of different and even uncalibrated cameras. The BBA can take into account all the non-linearities and enables to achieve the best final accuracies.

The effects of the vegetation on any reconstructed surface can be mitigated by following a procedure based on the application of slope detection algorithms and SOR filters. This allows to minimize the influence of these factors on the subsequent DoD analysis. The use of a NRTK GNSS technique speeds up the survey of the GCPs needed for the georeferencing of dense clouds, DEMs, and orthomosaics, with accuracies comparable with the same precision achievable with the SfM approach. Although particular care should be taken for both on-field operations to prevent data biases, e.g., for the sinking of the pole’s tip during the survey, and for the validation stage of the products, the entire process may be applied also from non-specialist geomatic staff, since its workflow is not particularly difficult.

The results of this work confirm both the reliability and the affordability of the SfM approach by UAVs and indicates that it is a reliable tool for the interpretation of the change over time of very small shapes by the geologists.

The commercial availability of both lightweight UAVs, often very easy to operate, and software implementing SfM algorithms will expand the use of these techniques, not only for coastal environments but wherever rapid acquisition is needed, and high spatial resolution must be achieved. Many types of applications can be already found in the scientific and technical literature, from the mapping of coastal areas [25] to the detection of river morphology [26], from the monitoring of melting glaciers dynamics [27] to the assessment of landslides [28].

Further developments of this research may be imprinted to exploit the multispectral data acquired by the Sentera Single sensor shown in Figure 5c and likely using more recent spectral sensors in the future to support the develop even more accurate filtering procedures. In fact, some small residual effects caused by vegetation are still recognizable in some regions of the models, especially approaching to the back-dunes. The use of an on-board NRTK may also certainly speed up the survey, avoiding the deploying of a high number of GCPs, while small LiDAR on-board instrumentations may also represent an interesting alternative technique. The application of Digital Image Correlation (DIC) techniques to the imagery datasets to quantitatively trace dune progradation may also represent an interesting technique for further geomorphological quantification of the progression phenomenon. Indeed, the DIC has been applied successfully for monitoring the migration rates of subarctic dune fields [29] and active landslides [30].

Despite a variety of many other alternative surveying methods, the digital photogrammetry represents, at present, an excellent technique able to achieve both high spatial and temporal resolution at a truly affordable cost.

## Figures and Tables

**Table 1:** Aircraft and camera specifications.

Aircraft Specifications			
		***DJI Phantom 2 Vision***	***DJI Phantom 3 Professional***
Take-off weight		1160 g	1280 g
Max flight speed		15 m/s	16 m/s
Max flight time		16 min	18 min
Hovering accuracy	Horizontal	±2.5 m	±0.3÷1.5 m
	Vertical	±0.8 m	±0.1÷0.5 m
**Cameras Specifications**			
		***Panasonic Lumix DMC–GM1***	***DJI FC300X***
Sensor format		17.3 mm ×13.0 mm	6.5 mm ×4.9 mm
Focal length		12 mm	3.6 mm
35 mm equiv. focal length		24 mm	20 mm
Image resolution		4592×3448	4000×3000
Field of View		72°	84°
Pixel size		3.8 μm	1.6 μm
GSD at 40 m altitude		1.3 cm	1.8 cm

**Table 2:** Flight plan specifications.

Flight Plans Specifications	
Altitude	40 m
Longitudinal overlap	80%
Side overlap	60%
Speed of aircraft	ca. 3 m/s
Exposure time	1/400 ÷ 1/1000 s
ISO sensitivity	100 ÷ 400

**Table 3:** Summary of the residuals.

Residuals		Survey Date					
		November 2015	March 2016	June 2016	November 2016	May 2017	December 2017
**East**	*RMSE* [m]	0.029	0.011	0.023	0.044	0.036	0.052
	*Min* [m]	−0.058	0.024	−0.096	−0.068	−0.102	−0.078
	*Max* [m]	0.050	0.020	0.043	0.050	0.054	0.107
**North**	*RMSE* [m]	0.018	0.016	0.026	0.02	0.037	0.032
	*Min* [m]	−0.037	−0.031	−0.102	−0.036	−0.069	−0.061
	*Max* [m]	0.03	0.027	0.034	0.045	0.090	0.057
**Up**	*RMSE* [m]	0.006	0.003	0.032	0.040	0.024	0.011
	*Min* [m]	−0.010	−0.006	−0.055	−0.041	−0.054	−0.027
	*Max* [m]	0.008	0.003	0.032	0.040	0.038	0.019
**3D**	*RMSE* [m]	0.035	0.019	0.036	0.053	0.057	0.062
	*Min* [m]	0.011	0.005	0.013	0.029	0.009	0.018
	*Max* [m]	0.063	0.032	0.142	0.073	0.104	0.121

**Table 4:** Zonal statistics on the selected DoD regions.

Zone Classification	Pixel Count	Mean [m]	Std. Dev. [m]	Min [m]	Max [m]
stable dunes	92,662	−0.029	0.046	−0.190	0.269
zone *A*	37,350	0.229	0.055	0.000	0.433
zone *B*	43,610	−0.116	0.076	−0.412	0.126
zone *C*	43,851	0.359	0.131	−0.052	0.716

## Abbreviations

The following abbreviations are used in this manuscript:

BBA	Block Bundle Adjustment
CORS	Continuously Operating Reference Stations
DEM	Digital Elevation Model
DIC	Digital Image Correlation
DoD	DEM of Difference
ETRF	European Terrestrial Reference Frame
ETRS	European Terrestrial Reference System
Exif	Exchangeable image file format
GCP	Ground Control Point
GNSS	Global Navigation Satellite System
GPS	Global Positioning System
GSD	Ground Sample Distance
LiDAR	Light Detection And Ranging
NRTK	Network Real Time Kinematic
RDN	Rete Dinamica Nazionale (Dynamic National Network)
RGB	Red Green Blue
RMSE	Root Mean Square Error
SfM	Structure-from-Motion
SOR	Statistical Outlier Removal
TLS	Terrestrial Laser Scanner
UAV	Unmanned Aerial Vehicle

## References

[1] Short A., Hesp P. (1982). Wave, beach and dune interactions in southeastern Australia. Mar. Geol..

[2] Hesp P. (2002). Foredunes and blowouts: Initiation, geomorphology and dynamics. Geomorphology.

[3] Davidson-Arnott R. (2010). Introduction to Coastal Processes and Geomorphology.

[4] Hesp P.A., Walker I.J., Chapman C., Davidson-Arnott R., Bauer B.O. (2013). Aeolian dynamics over a coastal foredune, Prince Edward Island, Canada. Earth Surf. Process. Landf..

[5] Alesheikh A.A., Ghorbanali A., Nouri N. (2007). Coastline change detection using remote sensing. Int. J. Environ. Sci. Technol..

[6] Lynch K., Jackson D.W., Cooper J.A.G. (2009). Foredune accretion under offshore winds. Geomorphology.

[7] Westoby M., Brasington J., Glasser N., Hambrey M., Reynolds J. (2012). ‘Structure-from-Motion’ photogrammetry: A low-cost, effective tool for geoscience applications. Geomorphology.

[8] Cramer M., Stallmann D. (2002). System Calibration for Direct Georeferencing. Int. Arch. Photogramm. Remote Sens. Spat. Inf. Sci..

[9] Fitzgibbon A.W. (2001). Simultaneous linear estimation of multiple view geometry and lens distortion. Proc. IEEE Comput. Soc. Conf. Comput. Vis. Pattern Recog..

[10] Clapuyt F., Vanacker V., Oost K.V. (2016). Reproducibility of UAV-based earth topography reconstructions based on Structure-from-Motion algorithms. Geomorphology.

[11] James M., Robson S., d’Oleire Oltmanns S., Niethammer U. (2017). Optimising UAV topographic surveys processed with structure-from-motion: Ground control quality, quantity and bundle adjustment. Geomorphology.

[12] Jaud M., Passot S., Allemand P., Le Dantec N., Grandjean P., Delacourt C. (2018). Suggestions to Limit Geometric Distortions in the Reconstruction of Linear Coastal Landforms by SfM Photogrammetry with PhotoScan® and MicMac® for UAV Surveys with Restricted GCPs Pattern. Drones.

[13] Drummond C.D., Harley M.D., Turner I.L., A Matheen A.N., Glamore W.C. UAV applications to coastal engineering. Proceedings of the Australasian Coasts & Ports Conference 2015: 22nd Australasian Coastal and Ocean Engineering Conference and the 15th Australasian Port and Harbour Conference.

[14] Gonçalves J.A., Henriques R. (2015). UAV photogrammetry for topographic monitoring of coastal areas. J. Photogramm. Remote Sens..

[15] Scarelli F.M., Sistilli F., Fabbri S., Cantelli L., Barboza E.G., Gabbianelli G. (2017). Seasonal dune and beach monitoring using photogrammetry from UAV surveys to apply in the ICZM on the Ravenna coast (Emilia-Romagna, Italy). Remote Sens. Appl. Soc. Environ..

[16] Taddia Y., Corbau C., Zambello E., Russo V., Simeoni U., Russo P., Pellegrinelli A. (2017). UAVs to assess the evolution of embryo dunes. Int. Arch. Photogramm. Remote Sens. Spat. Inf. Sci..

[17] Mancini F., Dubbini M., Gattelli M., Stecchi F., Fabbri S., Gabbianelli G. (2013). Using Unmanned Aerial Vehicles (UAV) for High-Resolution Reconstruction of Topography: The Structure from Motion Approach on Coastal Environments. Remote Sens..

[18] Harwin S., Lucieer A. (2012). Assessing the Accuracy of Georeferenced Point Clouds Produced via Multi-View Stereopsis from Unmanned Aerial Vehicle (UAV) Imagery. Remote Sens..

[19] Cook K.L. (2017). An evaluation of the effectiveness of low-cost UAVs and structure from motion for geomorphic change detection. Geomorphology.

[20] Turner I.L., Harley M.D., Drummond C.D. (2016). UAVs for coastal surveying. Coast. Eng..

[21] Casella E., Rovere A., Pedroncini A., Stark C.P., Casella M., Ferrari M., Firpo M. (2016). Drones as tools for monitoring beach topography changes in the Ligurian Sea (NW Mediterranean). Geo-Mar. Lett..

[22] Wulder M.A., Hall R.J., Coops N.C., Franklin S.E. (2004). High Spatial Resolution Remotely Sensed Data for Ecosystem Characterization. BioScience.

[23] Agisoft (2018). Agisoft Photoscan User Manual, Professional Edition, Version 1.4. https://www.agisoft.com/pdf/photoscan-pro_1_4_en.pdf.

[24] Hugenholtz C.H., Whitehead K., Brown O.W., Barchyn T.E., Moorman B.J., LeClair A., Riddell K., Hamilton T. (2013). Geomorphological mapping with a small unmanned aircraft system (sUAS): Feature detection and accuracy assessment of a photogrammetrically-derived digital terrain model. Geomorphology.

[25] Nikolakopoulos K.G., Kozarski D., Kogkas S. (2017). Coastal areas mapping using UAV photogrammetry. Earth Resour. Environ. Remote Sens. Appl..

[26] Watanabe Y., Kawahara Y. (2016). UAV Photogrammetry for Monitoring Changes in River Topography and Vegetation. Procedia Eng..

[27] Rossini M., Mauro B.D., Garzonio R., Baccolo G., Cavallini G., Mattavelli M., Amicis M.D., Colombo R. (2018). Rapid melting dynamics of an alpine glacier with repeated UAV photogrammetry. Geomorphology.

[28] Thiebes B., Tomelleri E., Mejia-Aguilar A., Rabanser M., Schlögel R., Mulas M., Corsini A. (2016). Assessment of the 2006 to 2015 Corvara Landslide Evolution Using A UAV-Derived DSM and Orthophoto.

[29] Necsoiu M., Leprince S., Hooper D.M., Dinwiddie C.L., McGinnis R.N., Walter G.R. (2009). Monitoring migration rates of an active subarctic dune field using optical imagery. Remote Sens. Environ..

[30] Mulas M., Corsini A., Cuozzo G., Callegari M., Thiebes B., Mair V. (2016). Quantitative Monitoring of Surface Movements on Active Landslides By Multi-Temporal, High-Resolution X-Band SAR Amplitude Information: Preliminary Results.

